# Correlation between equi-partition of aminoacyl-tRNA synthetases and amino-acid biosynthesis pathways

**DOI:** 10.1093/nar/gkaa013

**Published:** 2020-01-22

**Authors:** Akio Takénaka, Dino Moras

**Affiliations:** 1 Research Institute, Chiba Institute of Technology, 2-17-1 Tsudanuma, Narashino, Chiba 275-0016, Japan; 2 Faculty of Pharmacy, Shenyang Pharmaceutical University, Benxi, Liaoning 117004, China; 3 Department of Integrated Structural Biology, Institut de Génétique et de Biologie Moléculaire et Cellulaire (IGBMC) 1 rue Laurent Fries, Illkirch 67404, France; 4 Centre National de Recherche Scientifique (CNRS) UMR 7104, France; 5 Institut National de Santé et de Recherche Médicale (INSERM) U1258, France; 6 Université de Strasbourg, Illkirch, France

## Abstract

The partition of aminoacyl-tRNA synthetases (aaRSs) into two classes of equal size and the correlated amino acid distribution is a puzzling still unexplained observation. We propose that the time scale of the amino-acid synthesis, assumed to be proportional to the number of reaction steps (*N*_E_) involved in the biosynthesis pathway, is one of the parameters that controlled the timescale of aaRSs appearance. Because all pathways are branched at fructose-6-phosphate on the metabolic pathway, this product is defined as the common origin for the *N*_E_ comparison. For each amino-acid, the *N*_E_ value, counted from the origin to the final product, provides a timescale for the pathways to be established. An archeological approach based on *N*_E_ reveals that aaRSs of the two classes are generated in pair along this timescale. The results support the coevolution theory for the origin of the genetic code with an earlier appearance of class II aaRSs.

## INTRODUCTION

Translation of the genetic codes to the amino-acid sequence of proteins is one of the most important events in every organism. The universality of the genetic code suggests that the code was established in its present form prior to the emergence of LUCA, the Last Universal Common Ancestor. Several hypotheses have been proposed to explain its origin and evolution (1–4). The coevolution theory postulates that prebiotic synthesis was an inadequate source of all twenty protein amino acids, and therefore some of them had to be derived from the coevolving pathways of amino acid biosynthesis. The latter would then represent the dominant factor shaping the code. The coevolution theory finds more and more additional support (5,6).

In all present organisms the aminoacyl-tRNA synthetases (aaRSs) are responsible for defining the genetic code by pairing the correct amino acids to their cognate tRNAs. The aaRSs are believed to have origins that may predate the last common ancestor and as such can provide insights into the evolution and development of the genetic code (7). Their function is to attach the amino acid substrate to the CCA end of the cognate tRNA through a two-step reaction. To reveal the structural aspects of the reaction mechanisms several crystal structures of aaRSs in the apo state or in complex with substrates (amino acids, ATP and tRNA) have been solved and analyzed (8). A parallel investigation based on primary sequences analysis led to the partition of aaRSs into two classes based on mutually exclusive sets of sequence motifs (9). A functional correlation could be made that rationalized this unexpected discovery: Class I aaRSs charge the aminoacyl moieties at the 2’-OH of the terminal tRNA ribose while Class II enzymes charge their aminoacyl substrate at the 3’-OH. These are two different chiral centers which explain the necessity of the different structures for the related active sites. The experimental evidences for that functional and puzzling discrepancy were long available (10). Two main structural features corroborated the partition discovery. All Class I catalytic domains adopt the widespread Rossmann fold while Class II active sites exhibit a unique anti-parallel β-sheet fold with three signature motifs. Furthermore aaRS-tRNA interactions are also correlated so that Class I aaRSs bind to the minor groove side of the tRNA acceptor stem while Class II aaRSs bind the major groove side (11). The relationship between the two classes of aaRSs and the genetic code was then revisited (12). A model of co-evolution where the two classes would descend from opposite strands of the same ancestral gene was proposed by Rodin-Ohno (13,14). The earlier proposal for an operational RNA code for amino acids, associated to the acceptor stem helix was another suggestion of interest on the same line (15). A recent in depth analysis of the acceptor stems sustains and extends this hypothesis (16). The interaction with tRNA would then be the main driving force for the class separation (17).

Which class came first is still an open question. The Rossmann fold is one of the most ancient and functionally diverse protein folds (18). The existence of several major Rossmann-fold enzymes classes, with different cofactors and catalytic chemistries, suggests a divergence from a common pre-LUCA ancestor (19). The class II catalytic domains have a unique alpha-beta fold similar to that of Biotin Ligase, BirA (7). Their evolution can be reconstructed from three peptidyl-hairpins. The origin of class II aaRSs has then be postulated at the transition between a thioester world of peptides and the phosphoester world of polynucleotides (20,21). An earlier emergence of class II aaRSs was also proposed based on a correlation between aa biosynthetic pathways and aaRSs (22). Both classes bind ATP but so far the conformation observed in class II enzymes has been found in one other enzyme only (23). Backbone Brackets and Arginine Tweezers were identified as the most compact ATP binding motifs characteristic for each class and traced back to the proposed Protozymes and their more efficient successors, the Urzymes (24).

**Figure 1.**
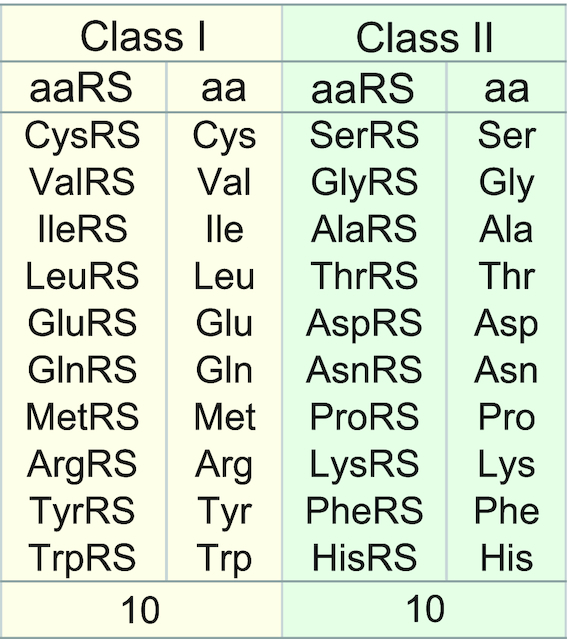
The two classes of aaRSs partitioned according to the structure characteristic of their active site domains and the corresponding aa.

While glyRS and alaRS are generally accepted as the best candidates for the origin of class II enzymes, the relative position in the order of appearance of the other aaRSs, notably the Class I members is not yet established. The paper addresses that question and the related one of the equal ratio of class members, referred to as equi-partition (Figure 1). In any archaeological approach, a time stamp is needed to track the related traces of fossils, ruins etc. In bio-molecular archeology, it is generally difficult to estimate the history of molecules because their traces are missing. As for the aaRSs, if the specificity for a given aaRS appears through aa mutations of the enzyme scaffold, it cannot exert its function without the proper aa substrate. Therefore aaRS appearance and substrate production should be correlated. The time lags of aa appearances can be expected to vary depending on the number of reaction steps or the number of enzymes involved. It is then possible to count the number of enzymes (*N*_E_) involved in the different pathways and correlate them to the time lags necessary for a given aa production. From that one might be able to construct correlations relevant to the origin of the respective aaRS. In this paper, we describe how to estimate each time lag and then examine whether a significant relationship exists between aa synthesis and aaRSs appearances.

## METHODOLOGY

### Simplified model of enzyme appearance

In the aa biosynthesis, individual aas are synthesized along their own pathway, in which several specialized enzymes (*X_i_*, *i* = 1, *N*_E_) are working through step-by-step reactions. For simplification, it is necessary to introduce the following assumptions:

1. Even if one of the members *X_i_* with a specific function has appeared, when *X_i_* has no target, it will be corrupted. When *X_i_* is adopted for some purpose, it will be able to exert its function, and the amount of *X_i_* will be magnified to maintain the homeostatic relation with other members.
2. To establish a pathway each *X_i_* must appear in the order of the reaction steps. Accordingly, the sum of each appearance time of all enzymes involved in the pathway could be regarded as the pathway formation time.
3. In the same way, it is assumed that the new aaRS corresponding to an aa will appear after the pathway formation. Therefore, when all aaRSs are arranged in the order of a time series of the pathway formations, it could suggest a timescale of aaRS appearances.

The time scale thus obtained may suggest a new perspective for the translation machinery appearance. The present study examines what features will emerge when using the present organism pathways.

The number of mutations can be linearly related to the time *D* spent for appearance of a new enzyme, and its appearance must be synchronized with the reaction steps. In the aa biosynthesis, when the number of concerned enzymes or reaction steps is defined as *N*_E_, it is possible to estimate the time *T* spent for the formation of the pathway by the following equation, *T* = *N*_E_ × *D*. If *D* is assumed to be constant, the value of *N*_E_ itself becomes a measure of the appearance time of the pathway. Their values should be proportional to the complexity or difficulty of biosynthesis reaction. Therefore, the estimated *N*_E_ value could be expected as a useful parameter in order for investigation of the bio-molecular archeology.

### Estimation of appearance time

**Figure 2.**
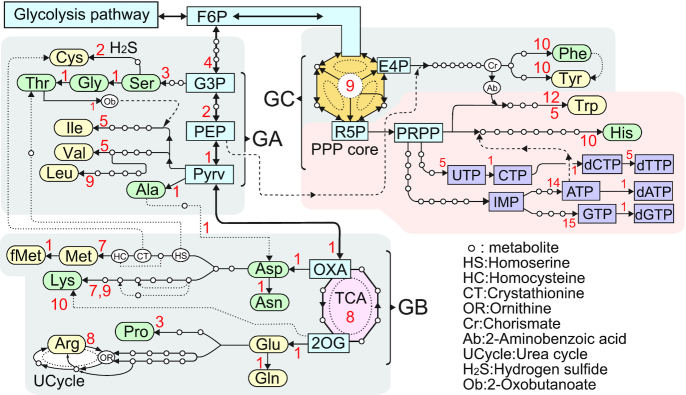
Biosynthesis pathways of amino acids and nucleotide triphosphates. Red colored digits indicate the number of reaction steps between the key metabolites. This diagram has been illustrated from the KEGG Pathway Database (25). Broken lines indicate supply of reactants. PEP is essential for making the aromatic benzene ring of Phe, Tyr and Trp. In addition, PRPP joins to form the indole moiety of Trp. While His is synthesized from PRPP with the help of ATP to make the imidazole moiety. Dotted lines may be for bypasses formed in later. All the aa pathways developed from F6P are divided into three groups; GA is on a nearest short path from F6P to Pyrv, GB is branched from TCA as a enzymatic circuit, and GC is based from the PPP core (see Figure 3B for details).

In order to evaluate the *N*_E_ values, all aa biosynthesis pathways have been retrieved from the Kyoto Encyclopedia of Genes and Genomes (KEGG) Pathway Database (25, see also http://www.genome.jp/kegg-bin/show_pathway?map01230) together with other related enzymes. A view of the pathways has been illustrated in Figure 2, in which the number of enzymes involved between the key metabolites is indicated as the local path (Δ*N*_E_*_s_*), ignoring the spontaneous reactions in the Δ*N*_E_ calculation. Because all the pathways are branched at fructose-6-phosphate (F6P) in the glycolysis, the common origin of the pathways is defined at F6P to compare the *N*_E_ values. Each *N*_E_ value is thus the sum of Δ*N*_E_*_s_* (*N*_E_ = Σ*_s_*Δ*N*_E_*_s_*, *s* = 1,number of local paths) on the pathway from F6P to the target aa. For example, the *N*_E_ value of the Thr pathway [F6P-G3P(glucose-3-phosphate)-Ser-Gly-Thr] is counted to be *N*_E_ = [4+3+1+1] = 9. In the same way, the Arg pathway [F6P-G3P-PEP(glucose-3-phosphate)-Pyrv(Pyruvate)-TCA (tricarboxylic acid cycle)-Glu-Arg] is *N*_E_ = [4+2+1+1+8+1+8] = 25 and the His pathway [F6P-PPP(pentose phosphate pathway)-His] is *N*_E_ = [9+10] = 19, etc.

## RESULTS AND DISCUSSIONS

### The biosynthesis pathways

**Figure 3.**
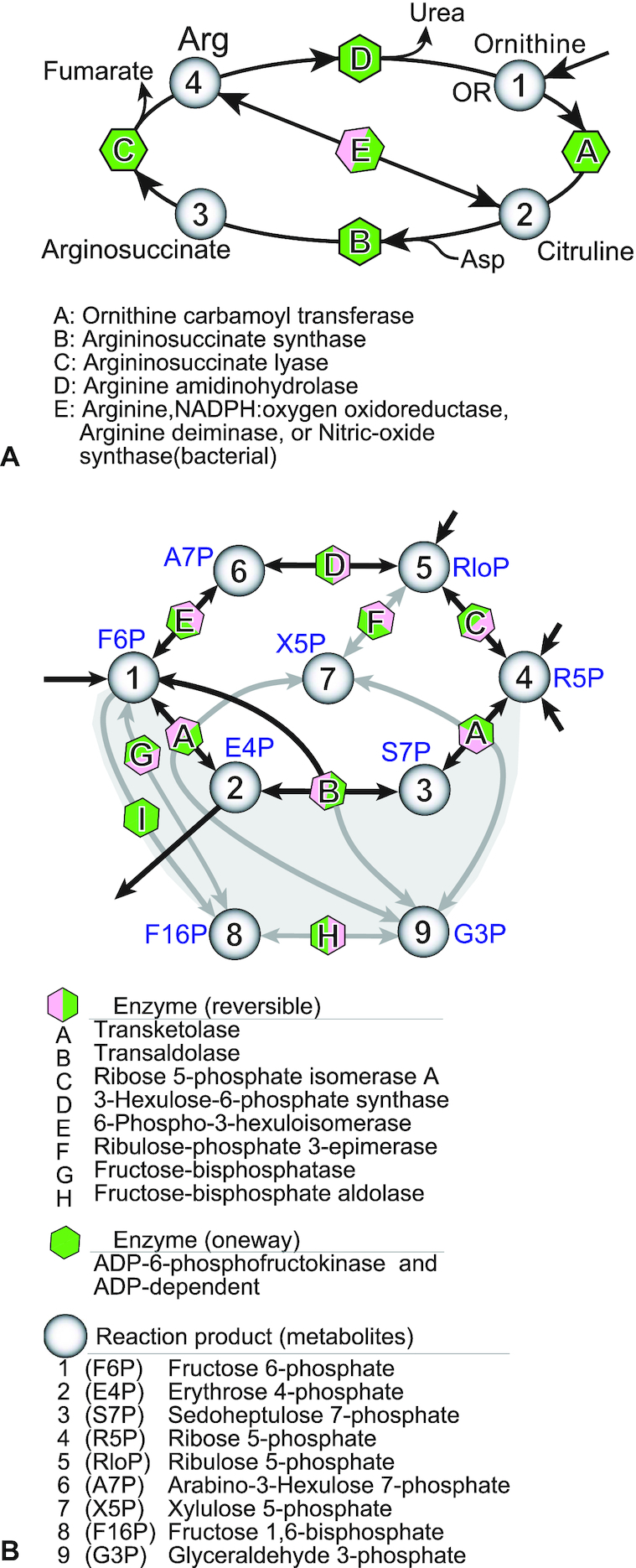
Two multiple circuits in the aa biosynthesis pathways. (**A**) UCycle formed at ornithin extended from Glu pathway. The triple circuits are composed of five enzymes to produce Arg and urea. (**B**) PPP (Pentose phosphate pathway) core composed of reversible reactions. The central hexagonal circuit is highly modified by adding several local paths to form a 3D architecture. This diagram has been illustrated from the biosynthesis pathway maps of the KEGG database.

**Figure 4.**
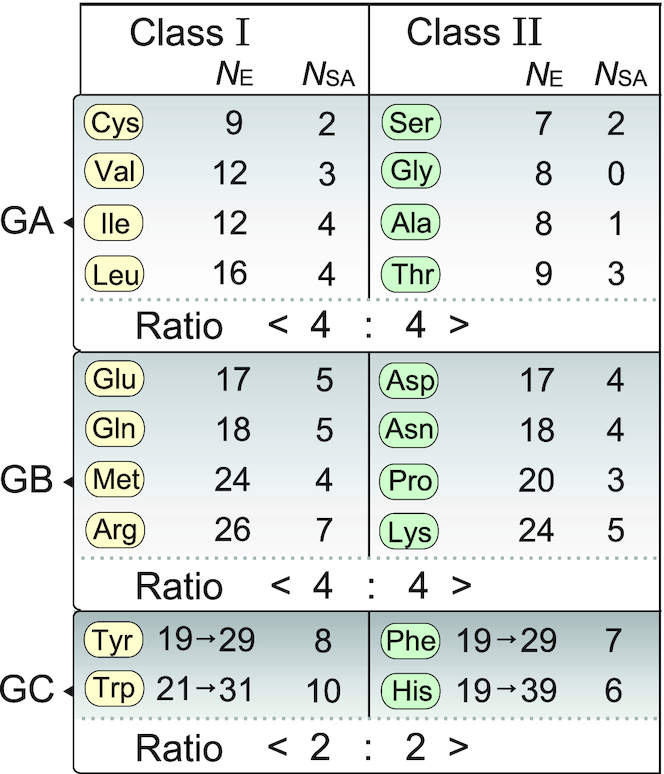
Amino acids rearranged according to the number of enzymes (*N*_E_) involved in the aa biosynthesis pathways. Amino acids are partitioned into the two classes to which the corresponding aaRSs belong, and divided into the three groups depending on the biosynthesis pathways (see Figure 2). *N*_SA_ indicates the number of the non-hydrogen atoms of aa side chain. The digits arrowed are the values of *N*_E_ compensated for the formation of multiple-reaction circuit. The ratios indicate the number of aaRSs in the box of each class.

Figure 2 shows a whole view of the pathways with the number of concerned enzymes or synonymously with the number of the local reaction steps (Δ*N*_E_*_s_*) between the key metabolites. The two reaction circuits of urea cycle (UCycle) and PPP core are shown in details in Figure 3A and B, respectively. As all the pathways are commonly passing through F6P, the origin of pathways has been defined at this metabolite. The total *N*_E_ values of all aas, calculated by the sum of the local Δ*N*_E_*_s_* from F6P to the target aa, are shown in Figure 4. A broken line indicates supply of PEP for making aromatic six-membered moieties of Phe, Tyr and Trp. While 5-phospho-α-D-ribose-1-diphosphate (PRPP) extended from R5P of the PPP core is an essential metabolite for the Trp and His biosynthesis. At the same time, this PRPP is the key station to develop the nucleoside-triphosphate (NTP) and its deoxy derivative (dNTP) biosynthesis pathways, as shown in Figure 2. Dotted lines were ignored as bypasses in the present analysis because they might appear later. All the pathways can be clustered in three groups (GA, GB and GC) depending on the architectural constructs (linear, single circuit and multi-circuit).

### Three characteristic groups of the aa biosynthesis pathways

The GA group which encompasses linearly from F6P to Pyrv is located at the beginning of a metabolic path after the glycolysis (see Figure 2). From the origin, the *N*_E_ values between F6P and Pyrv is counted to be seven by adding the four enzymes (6-phosphofructokinase 1, fructose-bisphosphate aldolase I, glyceraldehyde 3-phosphate dehydrogenase and phosphoglycerate kinase) from F6P to G3P, the two enzymes (2,3-bisphosphoglycerate-dependent phosphoglycerate mutase and enolase) from G3P to PEP and one enzyme (pyruvate kinase) between PEP and Pyrv. In this group, eight amino acids (Ser, Gly, Ala, Thr, Cys, Val, Ile and Leu) appear near the origin. These are limited to aas carrying small or medium hydrophobic groups or those with hydroxyl or thiol group.

The GB group extended from GA contains a TCA cycle which is an enzymatic circuit composed of eight kinds of enzymes (citrate synthase, aconitase, isocitrate dehydrogenase, α-ketoglutarate dehydrogenase, succinyl-CoA synthetase, succinate dehydrogenase, fumarase and malate dehydrogenase). Therefore, the TCA cycle (8-membered single circuit) formation from F6P contains 16 steps as the local path. And then from the two ports of Oxaloacetate (OXA) and 2-Oxoglutarate (2OG), another eight pathways are extended. Asp, Asn, Met and Lys belong to OXA, and Glu, Gln, Pro and Arg belong to 2OG. The GB group allows aas to contain rather large hydrophilic side chains (acidic, its amide and basic). As compared with GA, the complexity of pathways increases as shown in Met which requires thiolation of Asp and then methylation of the thiol group. In the Lys and Arg pathways, there is more than one pathway. Especially the Arg pathway requires the UCycle composed of 3-circuits with five different enzymes to prepare the guanidine moiety of Arg (see Figure 3A).

The third group GC is independent from GA and GB, so that it starts from the pentose phosphate pathway (referred to PPP core hereafter), attached directly to F6P adjacent to the glycolysis pathway. This core is formed to produce the remaining four kinds of aromatic aas, independently from the GA and the GB groups. Here the PPP core is defined as a group of enzymes which are linked together using reversible reactions. The central nine enzymes (A∼I) could form a core to produce nine kinds of metabolites (1–9), as shown in Figure 3B. In it the three different hexagonal circuits [1–2–3–4–5–6–1, 1–2–3–4–9–8–1 and 1–6–5–4–9–8–1] are fused together like the highly sophisticated 3D architecture. In addition, the top hexagon surrounded by black arrows in Figure 3B is further divided into three small circuits. Each circuit contains four members. This core is symbolized in Figure 2. All the enzymes except I (ADP-6-phosphofructokinase & ADP-dependent glucokinase) catalyze reversible reactions. In order to supply a sufficient amount of metabolite products to the four pathways, all enzymes must be controlled through reversible catalytic reactions. It suggests that they could be assembled as a complex. As shown in Figure 2, there are two key metabolites, erythrose-4-phosphate (E4P) and ribose-5-phosphate (R5P), which are linked to the GC dependent aa biosynthesis pathways. With the help of PEP, Phe and Tyr are synthesized from E4P. At the metabolite chorismate (Cr) of this pathway, the Trp pathway is branched, and by getting the help of PRPP the Trp pathway is completed. On the way from PRPP to His, ATP synthesized in the long ATP pathway (see Figure 2) is decomposed to supply the imidazole moiety as the side chain of His.

### Estimated *N*_E_ values

In Figure 4, the calculated *N*_E_ values are separated in three groups (GA, GB and GC) and partitioned into the two classes of aaRSs, and then aas are rearranged in the order of the *N*_E_ values as the timescale. Prior to the smallest amino acid Gly, Ser appears as the first aa through dehydrogenation-amination-dephosphorylation of G3P. After that, Ser is converted to Gly with the help of 5,10-methylenetetrahydrofolate. On the other hand, Ser is converted to Cys by acetylation and thiolation using hydrogen-sulfide from the sulfur metabolism. On the other hand, Ala is produced directly from Pyrv. From Pyrv, other three hydrophobic aas are also synthesized. It suggests that the side chain size is proportional to the *N*_E_ values. In other words, when the size of the side-chain is larger, its biosynthesis lag time is increased.

A similar trend can be seen for the GB aas, but the *N*_E_ values of GB become larger than those of GA, reflecting the difference in the side-chain size or the complexity between the two groups. In GB, Asp appears directly from OXA and then amidated to produce Asn. In the same way, Glu appears from 2OG and then it is converted to Gln. The *N*_E_ values are varied in parallel (correlatively) between the Asp and Glu pathways. Furthermore, the Met and Lys pathways are extended from Asp. The Lys pathway contains multiple paths. In a similar way, the Pro and Arg pathways are extended from Glu. As shown in Figure 3A, the Arg pathway contains UCycle to generate the guanidine moiety of Arg from ornithine. The correlation between the side-chain size and the *N*_E_ values described in GA is more apparent for these GB aas. This observation supports the proposition that the *N*_E_ value is reliable and useful to evaluate aa appearance.

In the GC pathways, the PPP is the basic core to produce all the four aromatic aas (Phe, Tyr, Trp and His), as shown in Figures 2 and 3B. The Δ*N*_E_ value of the core formation is estimated to be 9. Therefore, the *N*_E_ values of the Phe and Tyr pathways are estimated to be the same 19 for their similar size. The *N*_E_ value of Trp is increased reasonably for making the indole moiety on the phenyl ring. The His biosynthesis pathway is quite different from others so that it contains unique reactions, as described already. Because the imidazole moiety exhibits a neutral p*K*_a_ value, His residues can behave as essential partners of acidic aas for proton transfers catalytic reactions. However, the estimated *N*_E_ value of His is the same as those of Phe and Tyr and the all values are lower than those of GB. It may suggest that the necessity of time lag for the circuit formation of the complex machinery has to be included in the *N*_E_ calculation.

### Equi-partition of aaRSs at different aa appearance time

Figure 4 shows the two class partitioned aas rearranged along the estimated *N*_E_ values. In each class aas are divided into three groups according to their biosynthesis pathways. In GA, eight aas (Ser, Gly, Ala, Thr, Cys, Val, Leu and Ile) are partitioned along the *N*_E_ values. Four aas (Cys, Val, Leu and Ile) belong to class I and the remaining four (Ser, Gly, Ala and Thr) belong to class II. In GB also, four aas (Glu, Gln, Met and Arg) belong to the class I and the remaining four (Asp, Asn, Pro and Lys) belong to the class II. In the final GC, two aas (Tyr and Trp) belong to the class I and the remaining two (Phe and His) belong to class II. The equi-partition of aa biosynthetic pathways conserved in the three groups with different *N*_E_ values suggests that the equi-partition of aaRSs is correlated with aa appearance along the time scale.

### Correlation between equi-partition of aaRSs and aa biosynthesis

In this study, the time scale of aa appearance is estimated using the reaction steps of today's biosynthesis pathways. It is plausible that some other pathways were present earlier in evolution. Nevertheless most observed values show a reasonable correlation with the size of the side chains, reflecting the difficulty of biosynthesis. This means that the *N*_E_ value is a valid approach to evaluate the timescale of aa appearance. In the GC group, however, the *N*_E_ values seem to be underestimated. To compensate it, it is necessary to introduce an additional time cost such as the PPP core for the multi-enzyme machinery formation. To form a cyclic pathway, both ends of a linear reaction chain must be linked together. For this reason, an additional time lag (Δ*N*_A_) will be required for the circuit formation, such as TCA cycle and UCycle. In the case of PPP core, however, the three hexagonal circuits are fused together, as shown in Figure 3B. In addition, one of the hexagons contains three square circuits. Formation of such complicated multiple circuits will add large time lag.

From E4P of the PPP core (Figure 2), Phe and Tyr are synthesized with the help of PEP, essential for benzene (aromatic) moiety formation. While PRPP extended from the PPP core can be regarded as the essential key metabolite to produce the aromatic heterocycle of the side chains since all the bases (A, G, C, T and U) of NTP and dNTP are synthesized from this PRPP. Therefore Trp and His might be the last aas. Here it is interesting to compare the two aas. In Trp biosynthesis, PRPP reacts with 2-aminobenzoic acid from E4P to produce indole-glycerol, from which the released indole is bound to Ser. His side chain looks simpler, but the imidazole formation is difficult. The His pathway requires the adenine moiety of ATP as an reactant and the ATP biosynthesis pathway is longer than the Trp pathway. The resulting effects on *N*_E_ calculation is included in the compensation factor (Δ*N*_A_) of GC amino-acids.

It is difficult to accurately estimate the additional time lag (Δ*N*_A_). In order to form a circuit, each enzyme must be further modified by mutations so that it can collaborate with other enzymes working before and after the reaction series. In addition, it must be tuned to work repeatedly with high efficiency and to ensure the high quality. Here this time lag is assumed to be more than twice the Δ*N*_E_ value of the PPP core. So the Δ*N*_A_ value is roughly assigned to be 20. However, the pathways to Phe, Tyr and Trp extend from E4P just after F6P, suggesting that the three aas branch out before completion of the PPP core. Therefore, the Δ*N*_A_ values of these aas are assigned to be 10, half the Δ*N*_A_ value of the full PPP core formation. His is synthesized after the core formation with the help of ATP which is synthesized in the branch downstream. Therefore, the *N*_E_ values of the GC aas should be compensated by these additional time lags. By adding Δ*N*_A_ to the net *N*_E_ values of GC aas, we can obtain the following *N*_E_ values (*N*_E_ = 29, 29, 31 and 39 for Tyr, Phe, Trp and His, respectively), as shown in Figure 4.

**Figure 5.**
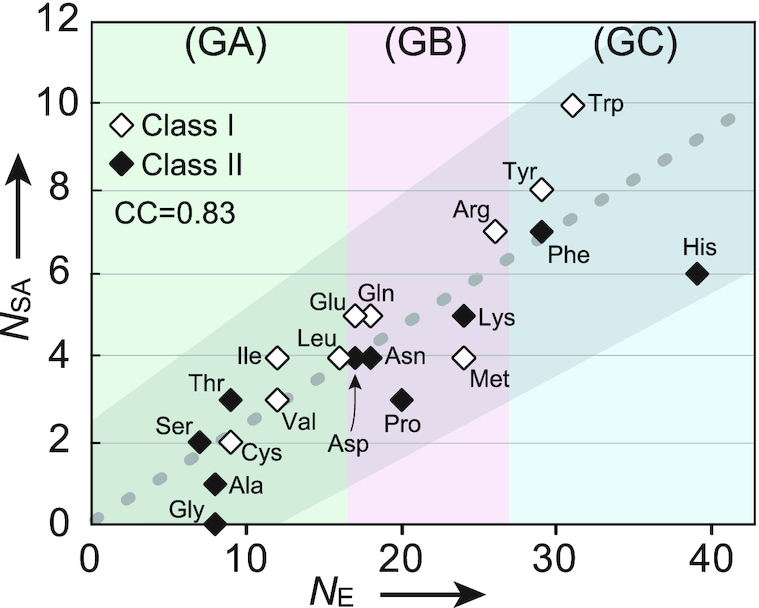
Graph showing the approximate linear correlation of *N*_E_ versus the number of aa side-chain atoms(*N*_AS_), which indicates high correlation between them throughout the three groups, suggesting the pair-wise appearance (co-appearance) of aaRSs from the two classes.

Figure 5 shows a plot of the side-chain size (number of atoms except hydrogen) along the *N*_E_ values throughout the three groups. The aas from the two classes are distributed along a same dotted line. The correlation coefficient containing both aas shows a high value (*CC* = 0.83 with *R*^2^ = 0.69 and *P*-value = 5.7 ×10^−6^), and even in each class, the *CC* value is 0.90 for class I and 0.87 for class II. This fact suggests that the compensation (*N*_A_) applied to the GC aas is well approximated. On the left side of the line Ser occupies the first position in front of Gly and Ala, because Ser can be easily prepared from glycolysis metabolite G3P. At the other end, Trp and His deviate from the line in two directions opposite to each other. Although Trp has a largest side chain, the indole moiety is prepared with the help of PRPP. The imidazole moiety formation requires the helps of additional enzymes and metabolites, as described above.

### Structural considerations

**Figure 6.**
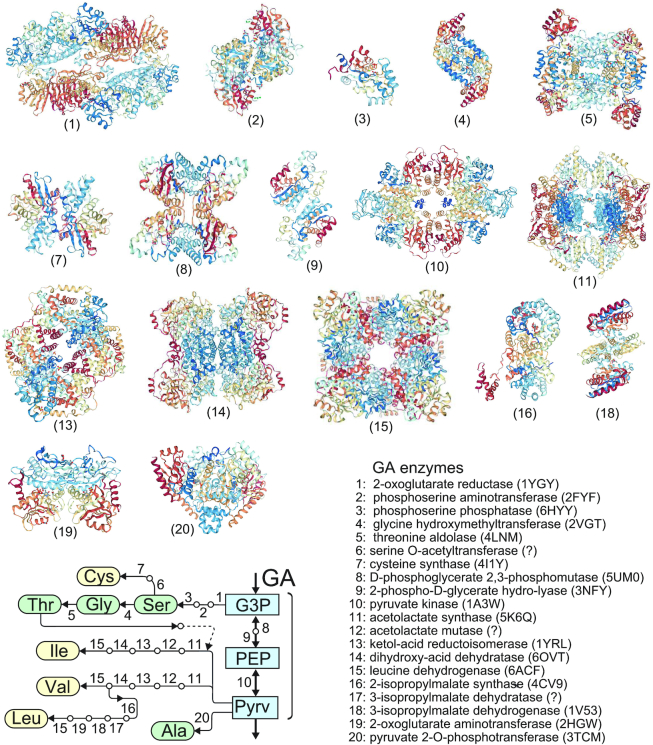
Structural features of the enzymes involved in the aa biosynthesis pathways which belong to GA, and the enzyme locations. There is no homologues. The PDB codes of X-ray structures are shown in parentheses. A symbol ? means the structure is unknown.

The molecular clustering of the enzymes along the GA pathway could suggest a common ancestry. To confirm that, the molecular structures of all the enzymes on the pathways of the GA group were examined for similarity in the folding and in the higher order architecture (Figure 6). Only PurL and PurM in the PuTP biosynthesis pathway are homologous (26,27). Furthermore, it seems that the relative amounts of Ile and Val are controlled by supply of Thr in the first reaction of Pyrv by acetoacetate synthetase, as shown in Figure 6.

Few exceptions to the functional and structural properties associated to the class partition can be observed, *i*.*e*. the existence of a class I lysRS instead of the canonical class II enzyme. The class I active site domains exhibit the Rossmann fold, an ancient pre-LUCA and highly abundant protein motif that could easily have adapted to different aa substrates. The existence in few archea of a class I lysRS could be explained by the long and flexible side chain, easily adaptable to the class I catalytic domain. See (7) for a deeper analysis of the evolutionary implications. Note that the coexistence of two lysRS in the same cell has not been observed.

The crystal structures of both TyrRS-tRNA^Tyr^ and TrpRS-tRNA^Trp^ show that the complex is a dimer and the binding mode of the tRNA somewhat similar to that of class II (28,29). In both cases, the symmetry of the enzyme dimers is however different from that of class II enzymes. The relative positions of the active sites with respect to the 2-fold axis explains the different tRNA binding modes. According to the timescale these enzymes are the last enzymes of the class I family to appear. A reconstruction of a pre-LUCA aaRS ancestor confirms the late addition of Trp to the genetic code (30). The observed differences could be the result of evolutionary constraints on the oligomeric structure of the protein. Note that structural and sequence comparisons suggest that this novel tRNA binding mode and recognition mechanism is very likely shared by other archaeal and eukaryotic TrpRSs, but not by bacterial TrpRSs (29).

GlyRs provides another example of polyphyletic origin with a resulting partition of class II enzymes into four sub-groups (a–d), bacterial GlyRS being part of subclass II (d) together with AlaRS (31). These different origins do not affect the ATP and aa binding mode. Whether the tRNA binding modes are affected remains to be seen but is unlikely since the dimeric structure of the active site domains is maintained.

PheRS chiral specificity is another puzzling observation. Unlike all other class II enzymes PheRS charge the aa to the 2’ hydroxyl group of the tRNA terminal adenine. The crystal structure of the T. *thermophilus* PheRS-tRNA^Phe^ complex explains the 2’OH binding by a different relative position of the tRNA terminal adenosine (180° rotation) with respect to the canonical binding position of the intermediate adenylate (32). When compared to other class II complexes, the shift of the bound tRNA may explain the rotation of A76 to another binding pocket. Like in the case of TrpRS the additional domains and/or the particular oligomeric structure of PheRS could be the cause of the shift. Note that mitochondrial PheRS and human PheRS differ markedly from heterodimeric eukaryotic cytosolic and bacterial analogs (33).

## CONCLUDING REMARKS

The first outcome of this study is that the equi-partition of aaRS between the two classes is kept at any times of the timescale through the three groups GA, GB and GC. It means that the aaRSs of the two different classes emerge in pairs along the timescale of aa expression. In order to accurately associate the proper aa and tRNA at the highest fidelity level, additional domains for editing, anticodon binding, *etc*, had to be attached. In order to construct the final most efficient aaRSs additional time lags were required. The maintained co-appearance despite these additional constraints indicates that the primary strongest pressure acts on the aa substrate selection.

An additional interest of the present results is that the *N*_E_ values of GA class II aas (Ser, Gly and Ala) are lower than those of class I (Figure 4), suggesting an earlier appearance of the corresponding aaRSs. Together with the unique characters of both their active site fold and the related ATP conformation the data favor the hypothesis of prior emergence of class II aaRSs.

Overall the results support the co-evolution theory and enlighten the role of aaRS-tRNA interactions in the process as well as the importance of the thermodynamic aspects and their strong link with metabolism. The latter had already been suggested to be important in linking the tRNAs modifications and the evolution of the genetic code (34).
